# Temperature Compensation for Reusable Piezo Configuration for Condition Monitoring of Metallic Structures: EMI Approach

**DOI:** 10.3390/s23031587

**Published:** 2023-02-01

**Authors:** Sushmita Baral, Prateek Negi, Sailesh Adhikari, Suresh Bhalla

**Affiliations:** 1 Department of Civil Engineering, Indian Institute of Technology (IIT) Delhi, New Delhi 110016, India; 2 Department of Civil Engineering, National Institute of Technology (NIT) Calicut, Kerala 673601, India; 3 Department of Civil Engineering, Pashchimanchal Campus, IOE, Tribhuvan University, Pokhara 33700, Nepal

**Keywords:** temperature compensation, piezo sensors, EMI technique, damage detection

## Abstract

This paper presents a novel algorithm for compensating the changes in conductance signatures of a piezo sensor due to the temperature variation employed in condition monitoring using the electro-mechanical impedance (EMI) approach. It is crucial to consider the changes in an EMI signature due to temperature before using it for comparison with the baseline signature. The shifts in the signature due to temperature can be misinterpreted as damages to the structure, which might also result in a false alarm. In the present study, the compensation values are calculated based on experiments on piezo sensors both in a free boundary condition and in a bonded condition on a metallic host structure. The values were further validated experimentally for damage detection on a large 2D steel plate structure. The variation in first natural frequency values for the unbonded piezo sensor at different temperatures has been used to develop the compensation algorithms. Whereas, in the case of the bonded sensor, the shift in structural peaks has been used. The developed compensation relations showed promising results in damage detection. Lastly, a finite element-based study has also been performed, supporting the experimental findings. The outcome of this study will aid in the compensation of the signatures in the structure due to temperature variation in the conductance signature.

## 1. Introduction

The structure health monitoring (SHM) of a civil structure is suggested to ensure its safety and proper functioning. Dreadful incidents in engineering structures have been reduced with the increase in SHM in damage detection. Generally, any damage begins from an early stage but may finally result in catastrophic loss over a period if not treated in time. Thus, it is necessary to determine the damage in its initial state to avoid further propagation. Therefore, regular inspection is carried out to safeguard the well-being of a structure, which is known as SHM. SHM is periodic monitoring of the structures from a safety point of view to identify, locate and determine the severity of the damages in the structures. It also includes the determination of the remaining life of the monitored structure. SHM gained popularity due to crucial capabilities such as identifying damage, assessing after retrofitting, validating design parameters, and monitoring external loads, stress distribution, deflection, and other factors on a structure. The structure is primarily monitored using two types of sensing technologies: conventional sensors and smart materials-based sensors. Conventional sensing technologies include electrical strain gauges, vibrating wire strain gauges, and accelerometers, while another type of sensing technology is based on intelligent materials. Smart materials are transducers whose behavior changes in a specific manner due to a particular type of stimulus input. Commercially, the two most common forms are Lead Zirconate Titanate (PZT) and polyvinylidene fluoride (PVDF) flexible composites. PZT patches have higher strength and stiffness than PVDF. PVDF is ductile and has shape conformability, whereas PZT is brittle and not acquiescent with curved surfaces.

The electro-mechanical impedance (EMI) technique was first developed by Liang et al. (1994) [1] and carried further by a number of researchers [2,3,4,5,6,7]. It is a non-destructive technique that uses piezo sensors for the condition monitoring of a host structure. A PZT patch acts both as an actuator and as a sensor. When it acts as the sensor, it functions in a direct mode in which it generates electric potential on applying stress. In the converse effect, the PZT patch produces stress when an electrical signal is applied across its surface. In recent years, piezo sensors have had significant applications in structural health monitoring, hydration monitoring of concrete, energy harvesting, and biomechanical applications [8,9,10,11]. This method adopts the study of the variation of admittance signatures with respect to the original admittance signatures from the piezo sensors–host structure combination.

Krishnamurthy et al. (1996) [12] found a decrease in the magnitude of the impedance peaks of a free PZT patch due to the increase in temperature. The temperature range of 25 to 75 °C was considered where the dielectric and piezoelectric properties of piezoceramic PSI-5A show a linear relation. However, it is nonlinear at a border temperature range. Normalization of the variation of impedance with temperature eradicates the effect of a change in the magnitude of impedance, which makes the variation independent of frequency. Changes in temperature, boundary condition, loading effects, etc., lead to a variation in the susceptance signature, whereas there is an insignificant change in the conductance signature. An insignificant change in the resistive portion of electrical impedance suggests the utilization of the real part of electrical admittance for the damage response, which minimizes the effect of temperature. 

The experiments conducted in a lab-controlled environment give stable results, but in an actual field condition, it is not possible due to the fluctuation of temperature [13]. The simulation study of each parameter controlling another parameter shows that the horizontal shift in the signature is due to a change in Young’s modulus of the structure, and the vertical shift is due to the change in electric permittivity of the PZT material (ε_33_) and piezoelectric strain coefficient (*d*_31_). Structural peaks are more affected rather than the PZT response peaks at various temperatures [14]. The shift of PZT resonance toward the left is mainly due to the softening of the bonding layer, structural properties, and piezoelectric properties of the PZT patch. Through simulation on FE software ANSYS, they validated that the shift of the signatures is caused by a reduction in stiffness of the bonding layer against increasing temperature. The variations in the amplitude of the impedance signatures were related to the temperature dependence of the capacitance of the piezoelectric sensor [15]. As a result of temperature variation, the shift in the resonance peak is not constant but increases with the increase in frequency. The frequency band used to calculate the damage indices played an essential role in compensating for temperature effects by maximizing the correlation coefficient. Na and Lee (2016) [16] performed an experimental investigation using the EMI technique in high-temperature pipeline facilities; it was concluded that damage identification is more successful above 200 °C, making the metal wire method suitable for the EMI technique in a high-temperature environment. Wandowski et al. (2016) [17] studied the effect of temperature in carbon fiber-reinforced polymer panels in the low-frequency range of 1–20 kHz, where a new approach for compensation of temperature influence on damage detection based on root mean square deviation index was proposed. Gianesini et al., (2020) [18] created the algorithm for compensation admittance signatures due to the temperature effect. The experiment was performed in the aluminum beam and steel pipe to minimize the impact of temperature variations on damage detection in the temperature range from −40 to 80 °C and the frequency range from 10 to 90 kHz. The variation of the admittance signatures due to the temperature effect in the civil, mechanical, and aerospace structures and the material was reported in several studies [19,20,21,22,23]. It was mentioned several times that there is a strong effect of temperature influencing the monitoring parameters. The studies have suggested a dependency on the temperature in the admittance signatures obtained from the EMI technique. 

The effectiveness of the EMI technique highly depends upon the surrounding conditions of the host structure. Still, compensation for temperature changes is skipped in regular practice. The temperature effect cannot be ignored in practical field conditions. The signature of the piezo sensors is very sensitive to temperature fluctuation, which triggers a false alarm. This paper tries to study the graduating effect of temperature on the EMI signatures of thin PZT patches in free and bonded conditions. Similarly, the compensation technique for the admittance signatures is also developed. The compensation of the temperature is calculated to the numerically obtained structural model. The results are further checked for the miniature aluminum beam sample and the large steel plate.

## 2. Basic Principle of EMI Technique

The primary aim of the SHM based on the EMI technique is to detect the damages that have occurred in the structures [18]. Equation (1) gives the governing equations for the complex electro-mechanical admittance of the PZT–structure coupled system based on Liang’s derivation.

(1) $$\bar{Y}=2\omega j\frac{wl}{h}\left[\bar{\varepsilon_{33}^{T}}+\left(\frac{Z_{a}}{Z+Z_{a}}\right)d_{31}^{2}\bar{Y_{0}^{E}}\left(\frac{\tan\kappa l}{\kappa l}\right)-d_{31}^{2}\bar{Y_{0}^{E}}\right]$$

where *ω* is the angular frequency, *j* is the imaginary number and is equal to $\sqrt{-1}$, *w* is the width of the PZT patch, *l* is the half-length of the PZT patch, *h* is the thickness of the PZT patch, $\bar{\varepsilon_{33}^{T}}\;$is the complex electric permittivity of the PZT material at constant stress, *Z_a_* is the actuator impedance, *Z* is the mechanical impedance of the structural system, *d*_31_ is the piezoelectric strain coefficient, $\bar{Y_{0}^{E}}$ is the complex Young’s modulus of the PZT patch at the constant electric field, and *k* is the wave number given by $k=\omega \sqrt{\frac{\rho }{\bar{Y^{E}}}}$. The impedance of actuator impedance is shown in Equation (2).

(2) $$Z_{a}=\frac{kh_{p}w\bar{Y^{E}}}{\tan kl\left(j\omega \right)}$$

In the EMI technique, the PZT patch, which is surface bonded or embedded in the host structure, is excited at a high frequency, in the range of 30 to 400 kHz, to generate the admittance. This technique generates unique admittance signatures, which are used as the baseline data. Any deviation in the signature with respect to the baseline data indicates the damage in the host structure. Due to excitation in high frequency, waves can scan the structure minutely, which is highly efficient at detecting the incipient damages in the structures. The deviation in admittance signatures may also occur due to the change in the temperature of the PZT patch and host structure instead of damage in the host structure. Relying on the admittance signatures, which are fluctuated by the temperature variation, may give a false alarm of the damage. The compensation algorithm is needed to address damage due to temperature change instead of the actual damage in the host structure.

Park et al. (1999) [24] found a significant horizontal and vertical shift in signatures due to a temperature in contrast to damage where the shifts are irregular. An empirical temperature compensation technique was developed, which can be applied to a complex structure. For the horizontal shift, an iteration was performed, whereas for the vertical shift, an empirical relation given in Equation (3) was developed.

(3) δv=∑i=1nRe(Yi,2)n−∑i=1nRe(Yi,1)n

where *δ_v_* is the vertical shift, *Y_i,_*_1_ is the original impedance at frequency interval *I* (baseline measurement), *Y_i,_*_2_ is the interrogated impedance at frequency interval *i* (subsequent measurement), and *n* is the number of data points measured. The compensation technique was validated on a bolted pipe joint, a gear, and a composite reinforced aluminum plate along with the experiments. 

## 3. Experimental Study on the Free Piezo Sensor and Miniature Aluminum Beam

**Figure 1:**
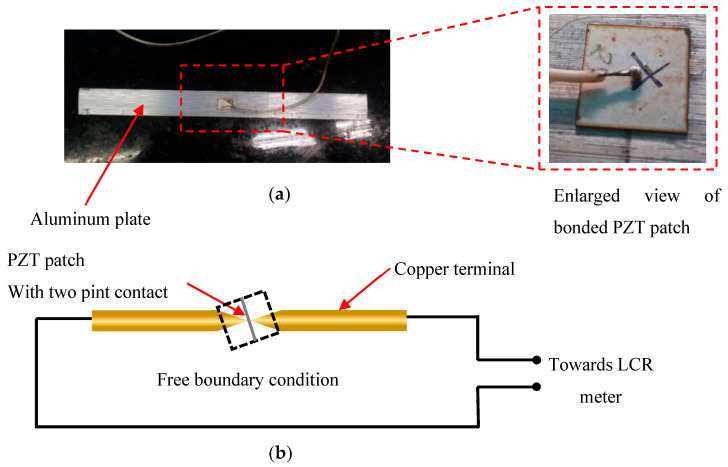
(**a**) Surface-bonded PZT patch on an aluminum plate, (**b**) free PZT patch setup for acquiring free piezo signatures.

This section presents the study of the effect on the admittance signatures of the piezo sensors when subjected to varying temperatures. The free piezo sensor was evaluated in the free and surface-bonded conditions. To investigate the temperature effect, a piezo sensor of (10 × 10 × 0.3) mm was taken. In the surface bonded condition, an aluminum plate of (200 × 25 × 2) mm was taken where the piezo sensor was bonded at the center of the beam, as shown in Figure 1a. The sensor was bonded using a two-part epoxy adhesive to obtain the signatures at different temperatures.

**Figure 2:**
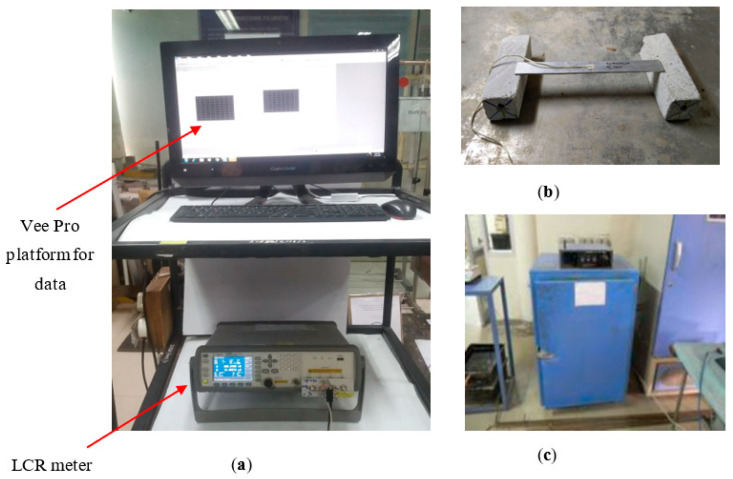
Experiment setup, (**a**) LCR meter and Vee-Pro platform, (**b**) setup for sensor bonded on the aluminum beam, and (**c**) oven used in the testing.

Similarly, a free piezo sensor of 10 × 10 × 0.3 mm was used to acquire signatures for the different temperature values. A single PZT patch was held between two pointed terminals, as shown in Figure 1b. Signatures were acquired through an Agilent E4980A LCR meter using the VEEPRO platform in a temperature range of 40–70 °C. The specimen with the bonded piezo sensor and the free piezo sensor was placed in the oven, and admittance signatures were acquired in the range of 1–200 kHz. Figure 2 shows the complete setup of the experiment. The deviation in the admittance signatures between the pristine signature and the temperature-affected signature was computed using the root mean square deviation (RMSD) index. Giurgiutiu and Rogers (1999) [25] defined the RMSD index as:

(4) $$RMSD\left(\%\right)=\sqrt{\frac{\sum_{j=1}^{N}{\left(G_{j}^{1}-G_{j}^{0}\right)}^{2}}{\sum_{j=1}^{N}{\left(G_{j}^{0}\right)}^{2}}}\times 100\;\%$$

where $G_{j}^{1}$ is the conductance after the temperature variation at a *j^th^* frequency and $G_{j}^{0}$ is the conductance at a pristine state at the same *j^th^* frequency, respectively. The individual effect of the surface-bonded piezo sensors and the free piezo sensors are discussed in the upcoming sub-sections. 

## 4. Experimental Results: Temperature Variation of Piezo Sensor and Host Structure

### 4.1. Effect of Temperature on Surface-Bonded Piezo Sensor

**Figure 3:**
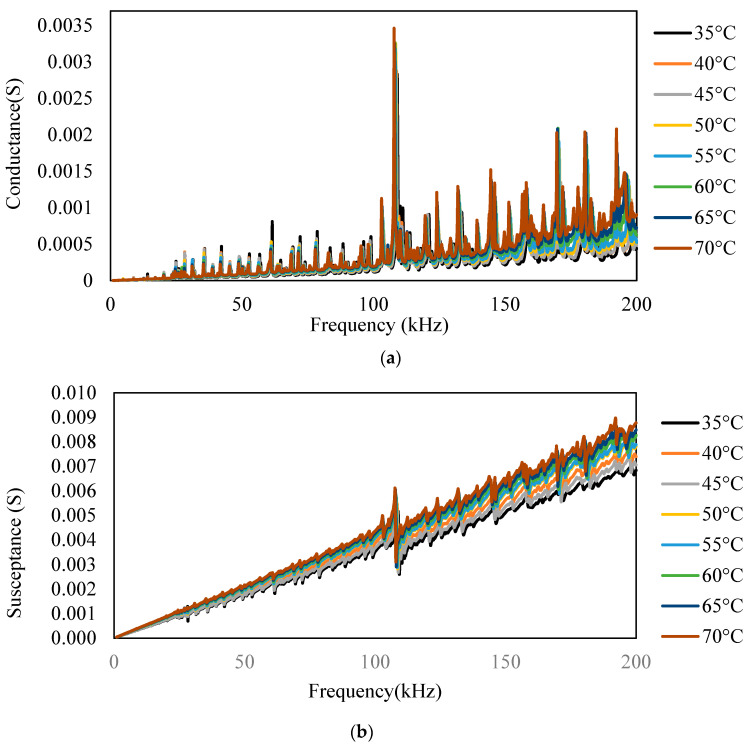
(**a**) Conductance and (**b**) susceptance signature of a bonded PZT patch measured at temperatures ranging from 35 to 70 °C.

**Figure 4:**
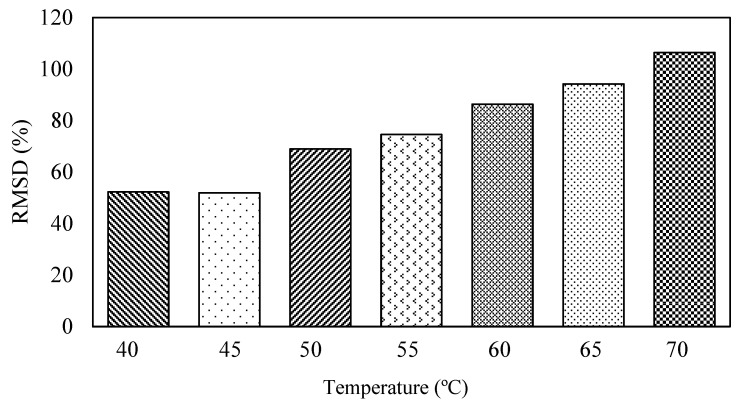
RMSD index at different temperature.

**Figure 5:**
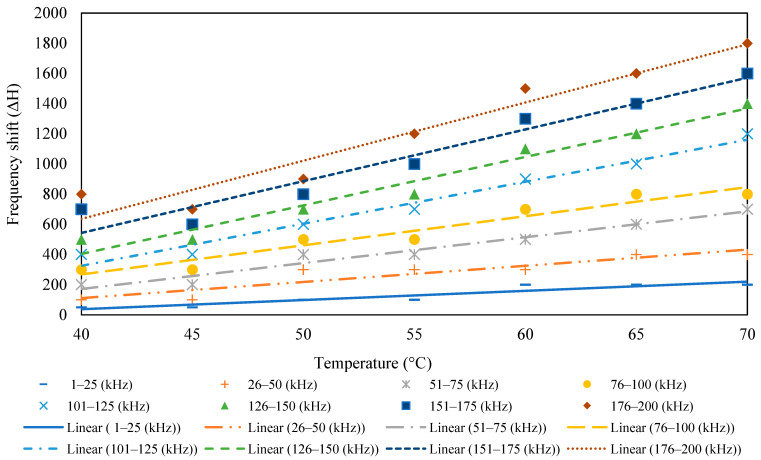
Horizontal shift as a function of temperature.

**Figure 6:**
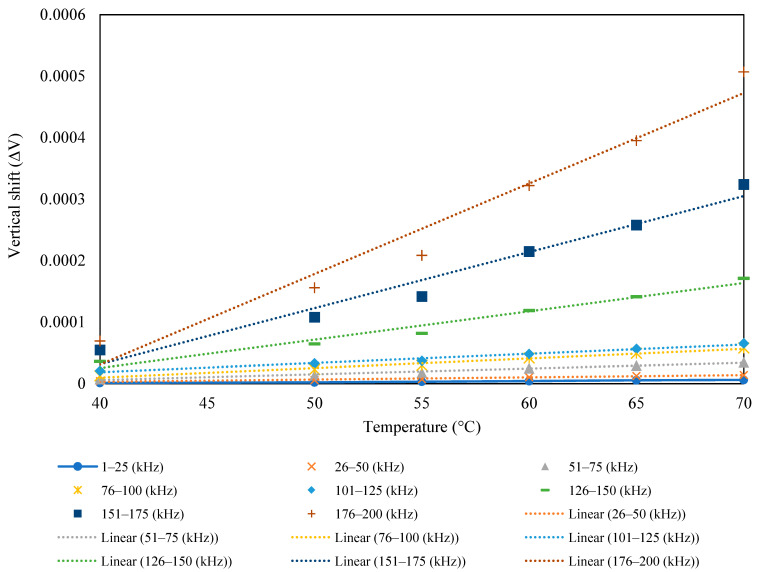
Vertical shift as a function of temperature.

The surface-bonded piezo sensor was placed in the oven maintaining temperatures from 35 to 70 °C. The admittance signature for the frequency range of 1–200 kHz is plotted for all the temperatures, as shown in Figure 3. For the surface-bonded piezo sensor, both the conductance and susceptance signatures shifted toward the upward-left direction with the increase in temperature, indicating a decrease in peak frequency and an increase in the admittance signatures. Figure 4 shows the RMSD index for different temperatures. It is observed that with an increase in temperature, there is an increase in the RMSD value. The horizontal shift was computed using iterations for different frequencies, and the vertical shift was calculated by taking the average of the difference between two signatures. Figure 5 and Figure 6 show the nature of the shift horizontally and vertically as the function of temperature. For the lower range of frequency, there is less change in the slope of the graph, whereas for the higher range of frequency, there is a higher value of the slope. This indicates that higher frequencies are more vulnerable to temperature change. It is observed that both horizontal and vertical shifts of the admittance signatures are temperature dependent. The magnitude of the change in shift of admittance signatures increases with an increase in temperature. The shift shows a linear variation with the increase in the temperature for all the frequency changes. The slope of the curve increases in both shifts, which indicates shifts are also frequency dependent. Based on these observations, a compensation technique for the signature has been developed, which is discussed in the latter part of this paper.

### 4.2. Effect of Temperature on Free Piezo Sensor

**Figure 7:**
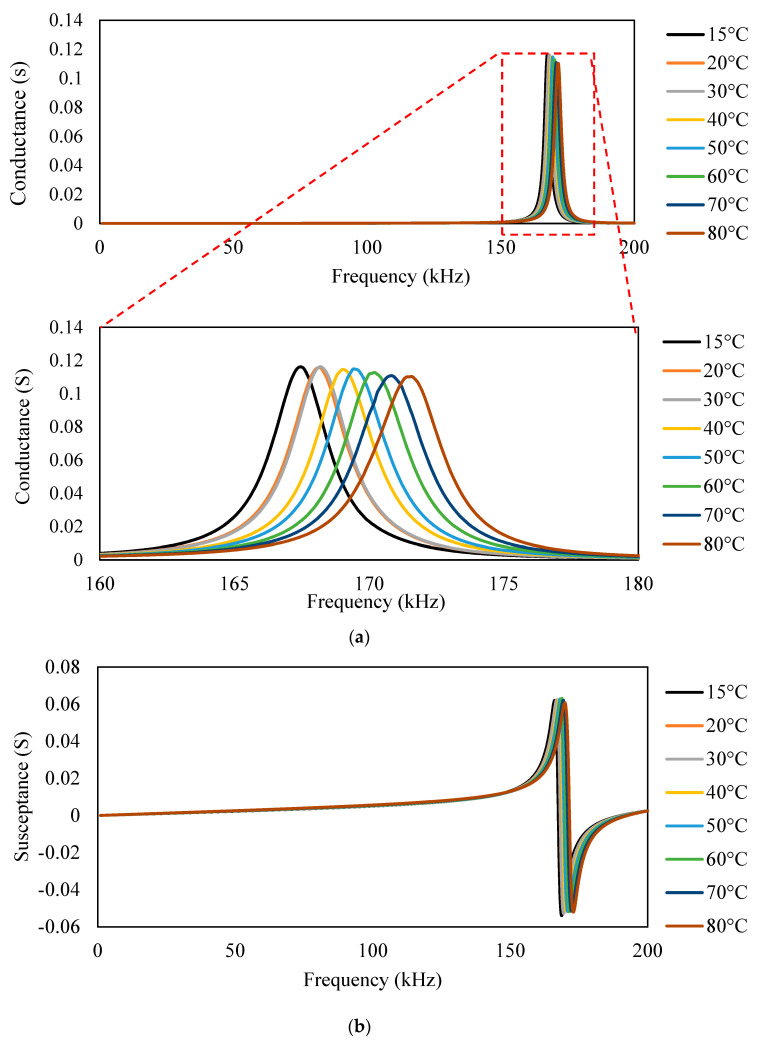
Free PZT sensor signatures at different temperatures: (**a**) conductance, (**b**) susceptance.

Figure 7 shows a plot of the conductance and susceptance signatures in the range of 1–200 kHz for the free piezo sensors. Unlike surface-bonded piezo, it was observed that in the case of free piezo sensors, both the conductance and susceptance signatures shifted toward the right with an increase in the temperature. The admittance signatures also shifted upward, although the upward shift was insignificant.

**Figure 8:**
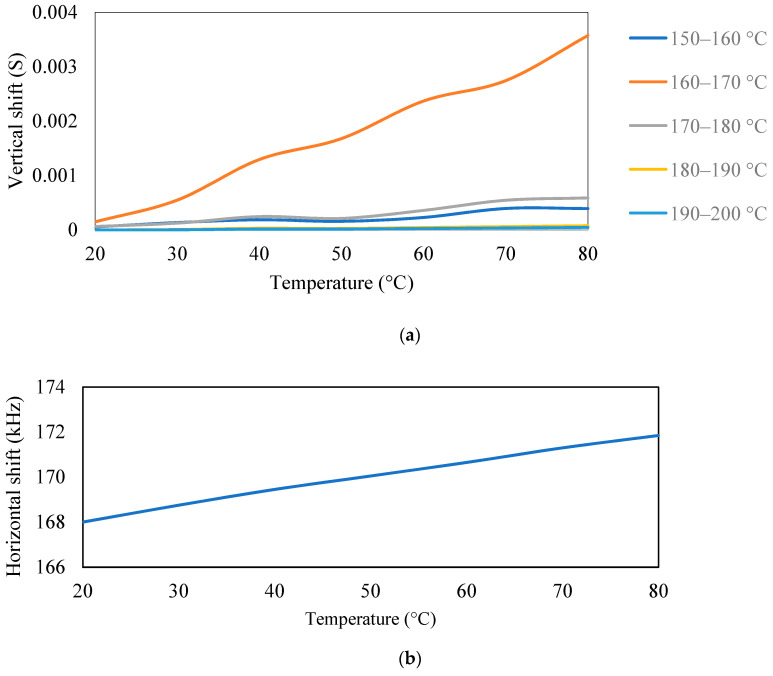
(**a**) Vertical shift; (**b**) horizontal shift as a function of temperature.

The vertical shift in the signature with the temperature variation is shown in Figure 8. The shift in the first natural frequency with respect to temperature has a linear distribution in horizontal and vertical shifts. 

### 4.3. Compensation for the Shift

**Figure 9:**
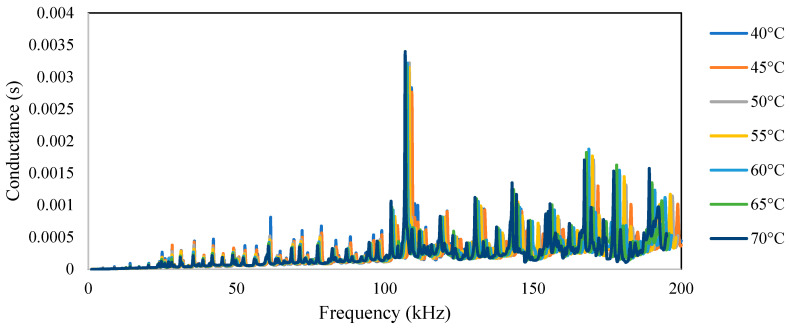
Compensated conductance signature for different temperature.

**Figure 10:**
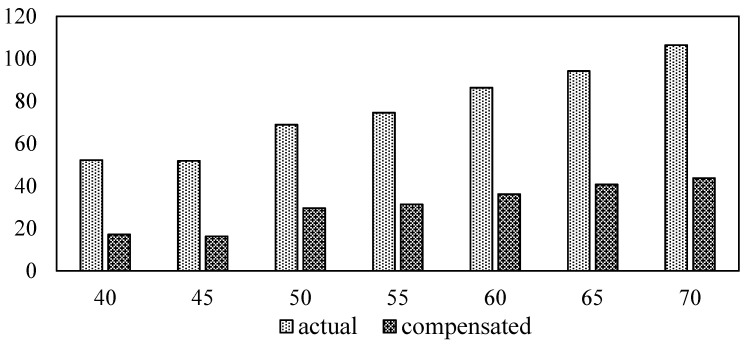
RMSD index plot of uncompensated and compensated signatures.

The shift in the signature is a function of the frequency range, as may be determined from the observation. For each frequency range, separate compensation equations were calculated. The admittance signatures were obtained from the experimental work on the PZT patch and host structures. The readings were plotted, and the deviation on the signatures was divided into the frequency range interval of 25 kHz. Then, the compensation equations were developed using regression analysis for the frequency range from 1 to 200 kHz. The compensation equations for the horizontal and vertical shifts are calculated and shown in Table 1. These factors were utilized to develop the compensation formulation. The results obtained after the compensation of different temperature is plotted in Figure 9. The RMSD index after compensation was computed as in Figure 10. It is observed that the values of the RMSD index decrease after the compensation of temperature. Failure to compensate for the admittance signatures may result in a false alarm of the damage. 

## 5. Numerical Modeling Using Coupled Field Analysis

**Figure 11:**
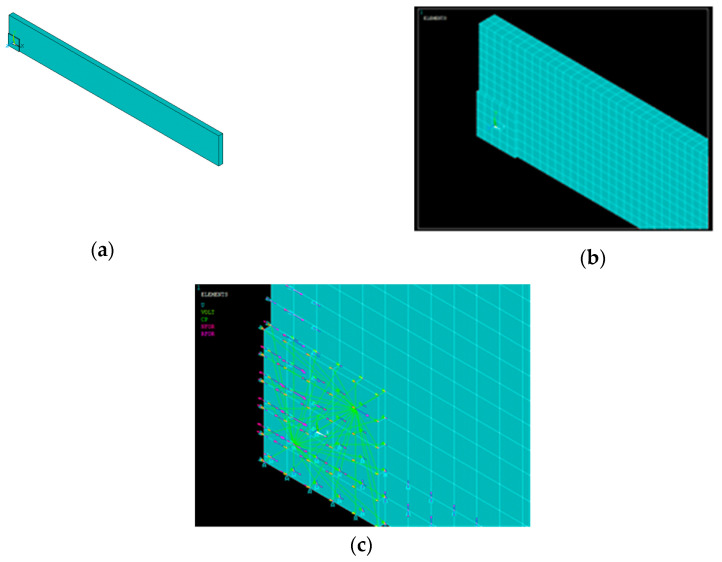
(**a**) 1/4th modeling of the aluminum block with PZT patch, (**b**) meshing of the model, (**c**) coupling effect.

A coupled field analysis with both electric and structural degrees of freedom was carried out to investigate the interaction of the piezoelectric sensor with the host structure, which reflects the electro-mechanical coupling feature of a piezoelectric material. The host structure in this coupled field investigation was an aluminum beam with a size of 20 × 25 × 2 mm on which a PZT patch of 10 × 10 × 0.3 mm was modeled. Only 1/4th of the specimen was modeled due to its symmetry on both axes. This reduced the significant computational time of the modeling [6,26,27]. Figure 11 shows the 1/4th modeling of the aluminum block with the PZT patch and its coupling effect on it. The key properties of the aluminum block are listed in Table 2. Young’s modulus, Poisson’s ratio, density, and Rayleigh damping coefficient have been considered in the FE modeling of the aluminum beam, as given in Table 3. Solid-45 and solid-5 elements were assigned to the aluminum beam PZT patch, respectively. A volume was created and meshed to obtain the admittance signatures numerically. The piezo properties were set according to the data given in Table 3. 

**Figure 12:**
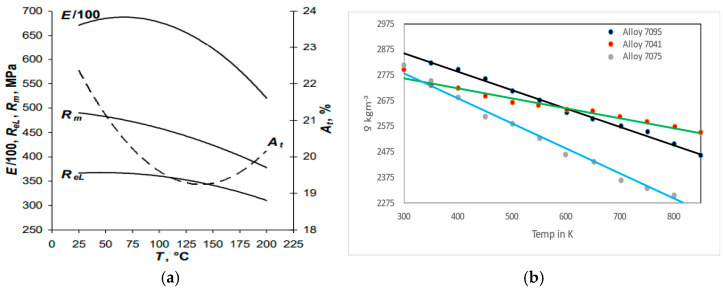
(**a**) Modulus of elasticity [29] and (**b**) density [30] of the aluminum beam at different temperature.

The boundary condition was assigned appropriately to the beam-piezo model. ANSYS follows the standard solid mechanics axis, whereas the PZT patch has the values in the IEEE axis format. The numerical model comprising the beam-piezo model was prepared by coinciding with the axes system. The detail of the axes system was previously reported by the authors [26]. In the model, the displacements along the x direction in the plane of symmetry of the host-piezo system running along the axes plane y-z were set to zero. Similarly, the displacements along the y direction were set to zero in the *x-z* plane of symmetry. The displacement along all the directions was set to zero in the *x-y* plane of the aluminum beam. The model was mesh with a global mesh size of 1 mm, for which mesh convergence was previously confirmed [4]. As the PZT patch was placed in the middle of the specimen, it was also modeled to 1/4th of its size. The nodes were then at the spacing of 1 mm; then, the load against the corresponding PZT dimensions was applied. The specimen was then excited to the frequency of 1–200 kHz. Figure 12a shows that the modulus of elasticity of the aluminum beam at different temperatures was considered. Similarly, the density of the aluminum beam (alloy 7041) at different temperatures was chosen as per Figure 12b.

**Figure 13:**
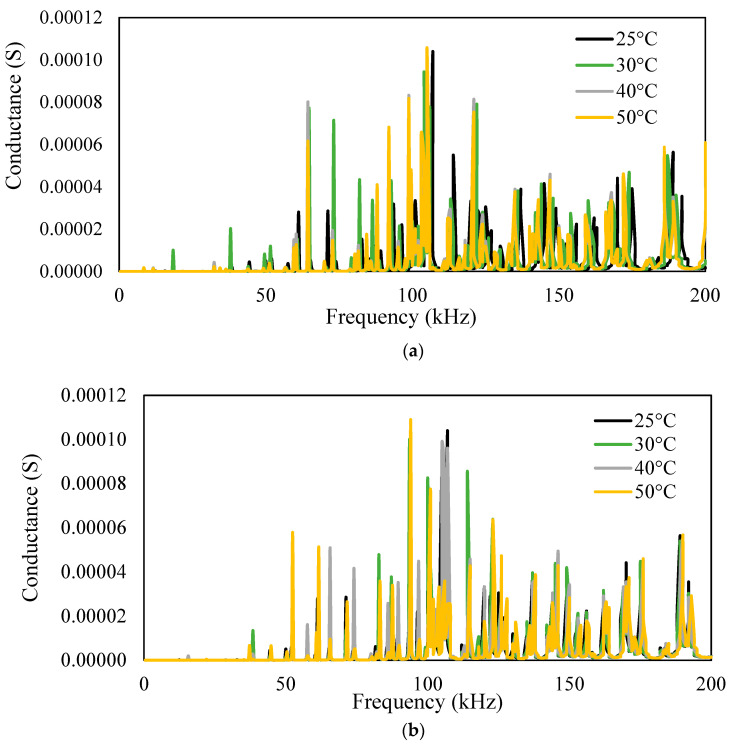
Conductance signature plot at different temperatures by varying the (**a**) modulus of elasticity and (**b**) density (of the aluminum block) + PZT patch.

**Figure 14:**
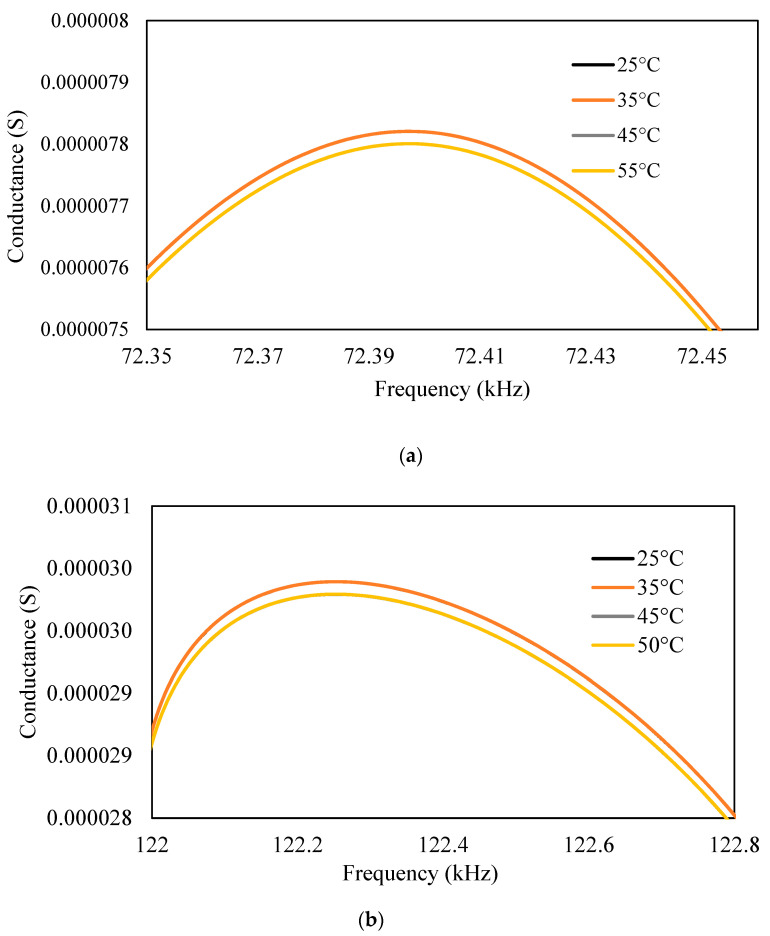
Conductance signature plot at different temperatures by varying (**a**) *ε*_33_ (**b**) *d*_31_ of PZT patch (of free PZT patch).

**Figure 15:**
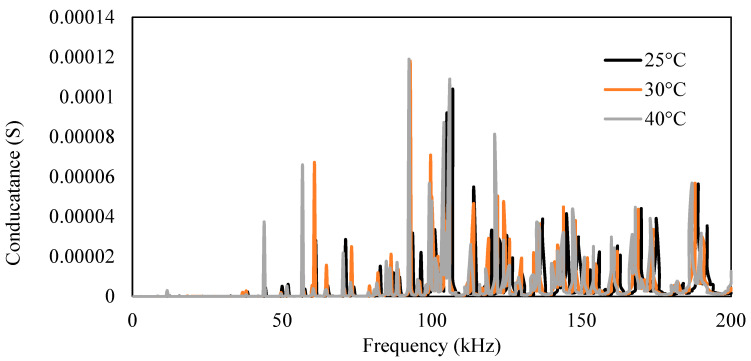
Conductance signature plot varying all the properties at different temperatures (of PZT patch + aluminum beam).

For the different temperatures, the physical properties, i.e., modulus of elasticity and density of aluminum, were varied to obtain admittance signatures in the range of 1–200 kHz, as shown in Figure 13, Figure 14 and Figure 15. For the different temperatures, there will be a change in the modulus of elasticity of the structure, which leads to a shift of signatures to the left for an increase in the temperature. There is also variation in the density due to the temperature change. The conductance signatures have increased in magnitude due to the decrease in the temperature. The change in the conductance signatures can also be observed when there is a change in the *ɛ*_33_ and *d*_31_ of the PZT patch. There is an increase in the conductance signatures when there is a decrease in the temperature. However, the change in the magnitude is very low for these cases. When there is an effect of temperature in all the physical properties and material properties, viz. density, modulus of elasticity, *ε*_33_, and *d*_31_, then there is a shift of peak on the left—upwards for the increase in the temperature.

It was observed from the ANSYS modeling results that modulus of elasticity and density of structure cause both a horizontal and vertical shift in the admittance signature, whereas *ε*_33_ and *d*_31_ are responsible for the vertical shift. 

This section has presented a numerical simulation conducted to study the effect of temperature on conductance signatures. The study indicates a strong dependence of shifts on temperature and frequency for both bonded and free piezo sensors. Based on these observations, a new compensation technique shows a good matching result. The analytical and modeling observations showed *ε*_33_ that *d*_31_ causes a vertical shift in the admittance signature.

## 6. Validation of Temperature Compensation Algorithm

In the previous section, it can be observed that the temperature significantly influences the signature of the piezo sensor. The temperature variation may raise a false alarm for damage detection or monitoring of the structure. In this section, an algorithm has been developed to validate the compensation formulation using piezo sensors on the 2D structure. 

The proposed algorithm is the damage detection on the structure with compensation of the signatures due to the temperature effect. In the beginning, the signatures were acquired for the structure in undamaged condition at room temperature, i.e., 27 ± 2 °C. Later, the signatures for the undamaged case at higher temperatures, i.e., above 30 °C, were also acquired. The horizontal and vertical shift per °C temperature in the signature for the undamaged case has been calibrated. The signature at a temperature above 40 °C for the damaged case is also obtained. The compensation of the shift due to temperature on both the higher temperature for the damaged and undamaged case is computed. The RMSD index for the damaged case after compensation indicates the actual damage to the structure.

**Figure 16:**
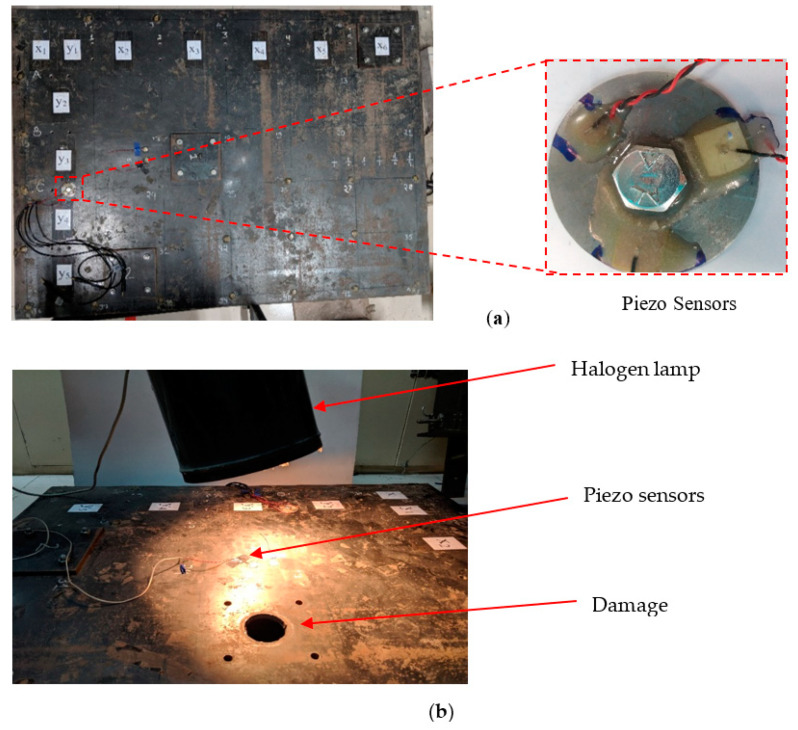
Experimental prototype structure: (**a**) steel plate with piezo sensors, (**b**) prototype structure with halogen lamp and damage.

The experimental specimen is a steel plate of size 1200 × 970 mm^2^, which is supported on box-type pipe of cross-section 38 × 38 mm^2^ and 3 mm thickness. Figure 16 shows the complete setup of the experiment. In the test specimen, piezo sensors developed in the same study were used as a specimen. Near the damage location at the center, piezo sensors were attached to the steel plate, and signatures were acquired for different temperatures. The temperature of the sensor and the area around the sensor were increased by a halogen lamp. The damage location was chosen, as shown in Figure 16. In pristine condition, the hole was covered by a plate of 150 × 150 mm in size. The plate was attached to the main plate by four bolts of 10 mm with 35 Nm torque.

The piezo sensors attached to the steel plate are electrically excited at the frequency range of 30–400 kHz with an impedance analyzer [31] (Agilent E4980A) to acquire an admittance signature. At room temperature, i.e., 27 ± 2 °C, signatures were acquired for the undamaged case. The halogen lamp temperature of the sensor and the area around the sensor were increased to 30 °C. Signatures were obtained at the same temperature for the undamaged case. The cover plate of 150 × 150 mm was removed from the main plate to create damage to the structure. The plate temperature was increased to 45 °C with the same halogen lamp for the damaged case. 

**Figure 17:**
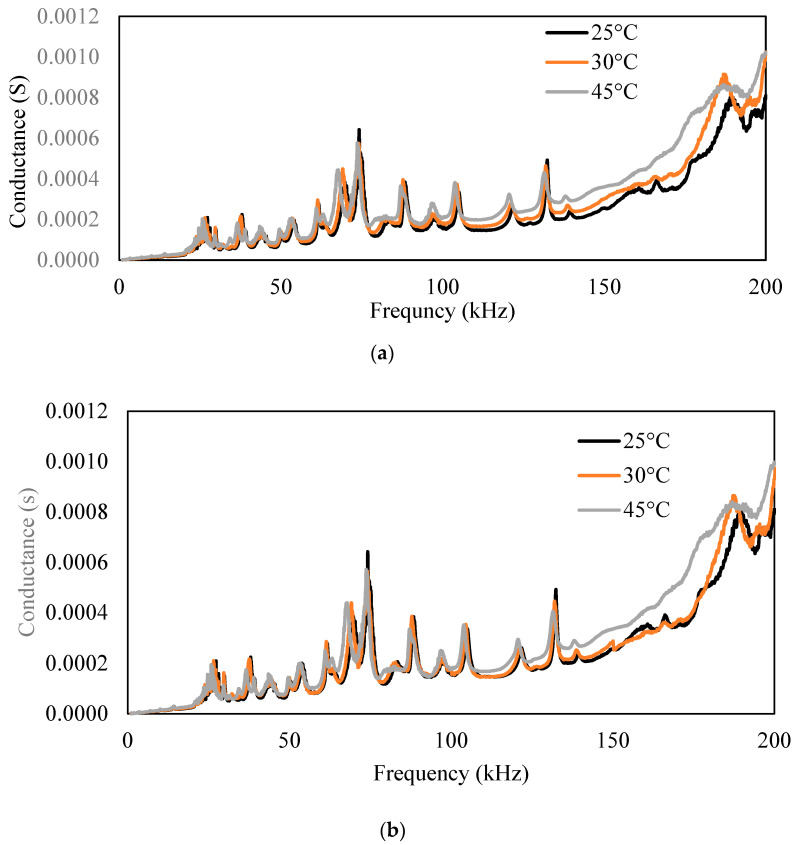
Conductance signature for the damaged and undamaged condition (**a**) with temperature effect and (**b**) after compensation of temperature effect.

As previously observed, with the increase in temperature, the conductance signature shifts toward the left and upward, as validated in the present scenario. In Figure 17, 25 °C and 30 °C readings are of the undamaged case, whereas 45 °C is the damaged condition. The RMSD index of the 30 °C (undamaged state) was 18.33%, and that of 45 °C (damaged state) was 35.01%.

Using the compensation formulation developed, the compensation of the signature was computed. Figure 17 shows the compensated signature. In Figure 17, the 25 °C plot is the actual signature, and the 30 °C plot is the compensated signature for undamaged conditions. The RMSD index calculated for the compensated signature was 11.93% for 35 °C, whereas for 45 °C, it was 28.93%.

This section validates the thermally compensated advanced reusable piezo sensor. A compensation formulation was valid on the piezo sensors with damage detection. The algorithm developed in this study can be implemented in a real-life structure where temperature variations are common. Steel bridges are the most common civil engineering structures which need monitoring and are prone to changes in the structure behavior due to temperature variation. The essential components of mechanical and aerospace structures are mostly made up of metallic structures. These structures are also heavily subjected to temperature variations. SHM of such structures by skipping the temperature effects may result in false data interpretation. The compensation algorithm developed in this paper can be implemented in metallic components of civil, mechanical, and aerospace structures where temperature effects are more pronounced. 

## 7. Conclusions

This study examined the effect of temperature change on the admittance signature of piezo sensors. The change in ambient temperature affects the admittance signatures, demanding the application of compensation to avoid false alarms of structural damage. The compensation of the recorded signatures helps determine the actual condition of the structure. It is necessary to compensate for the temperature of the admittance signatures at different frequency ranges, as the shift in the peaks of the admittance signatures has different magnitudes at each frequency range. Thus, the algorithm for the vertical and horizontal shift in admittance signatures at various frequency ranges was developed. The algorithm was derived successfully from the miniature specimen and validated for the large steel structure. The higher the temperature, the shift signatures are on the left side and upward. For lower temperatures, the variation of the admittance signatures is low. Thus, it was concluded that the higher frequency range was more susceptible to temperature change. The algorithms developed in this study can successfully be used to compensate for the admittance signatures of piezo sensors at different temperatures.

## Figures and Tables

**Table 1:** Compensation shift equation for the admittance signatures.

Frequency Range (kHz)	Horizontal Shift Equation	Vertical Shift Equation
1–25	6.0714 × T–205.36	2 × 10^−7^ × T–7 × 10^−6^
26–50	10.714 × T–317.86	4 × 10^−7^ × T–1 × 10^−5^
51–75	17.143 × T–514.29	1 × 10^−6^ × T–4 × 10^−5^
76–100	19.286 × T–503.57	2 × 10^−6^ × T–6 × 10^−5^
101–125	27.857 × T–789.29	2 × 10^−6^ × T–6 × 10^−5^
126–150	32.143 × T–882.14	5 × 10^−6^ × T–0.0002
151–175	34.286 × T–828.57	5 × 10^−6^ × T–0.0002
176–200	38.571 × T–907.14	5 × 10^−6^ × T–0.0002

**Table 2:** Properties of test specimen (aluminum beam at ambient temperature 27 °C).

Properties	Values
Modulus of elasticity (*E*)	68.95 GPa
Poisson ratio (*ʋ*)	0.33
Density (*ρ*)	2715 kg/m^3^
Mass damping factor (*α*)	0
Stiffness damping factor (*β*)	3 × 10^−9^

**Table 3:** Properties of piezoelectric conforming to grade PIC 151 [28].

Parameters	Symbols	Values	Unit
Density	*ρ*	7800	kg/m^3^
Dielectric Loss Factor	*tanδ*	0.02	-
Compliance	*S*_11_	15	×10^−12^ m^2^/N
	*S*_22_ = *S*_33_	19	
	*S*_12_ = *S*_21_	−4.5	
	*S*_13_ = *S*_31_	−5.7	
	*S*_23_ = *S*_32_	−5.7	
	*S*_44_ = *S*_55_	39	
	*S*_66_	49.4	
Electricity Permittivity	$\varepsilon_{11}^{T}$	1.75	×10^−8^ F/m
	$\varepsilon_{22}^{T}$	1.75	
	$\varepsilon_{33}^{T}$	2.12	
Piezoelectric Strain Coefficients	*d*_31_	−2.10	×10^−10^ m/V
	*d*_32_	−2.10	
	*d*_33_	5.0	
	*d*_24_	5.8	
	*d*_15_	5.8	

## Data Availability

Not applicable.

## References

[1] Liang C., Sun F.P., Rogers C.A. (1994). Coupled Electro-Mechanical Analysis of Adaptive Material Systems- Determination of the Actuator Power Consumption and System Energy Transfer. J. Intell. Mater. Syst. Struct..

[2] Ayres J.W., Lalande F., Chaudhry Z., Rogers C.A. (1998). Qualitative Impedance-Based Health Monitoring of Civil Infrastructures. Smart Mater. Struct..

[3] Abe M., Park G., Inman D.J. Impedance-based Monitoring of Stress in Thin Structural Members. Proceedings of the 11th International Conference on Adaptive Structures and Technologies.

[4] Bhalla S., Soh C.K. (2004). Structural health monitoring by piezo-impedance transducers. I: Modeling. J. Aerosp. Eng..

[5] Joshi B., Adhikari S., Bhalla S. Damage Sensitivity Investigations of EMI Technique on Different Materials through Coupled Field Analysis. Proceedings of the Sensors and Smart Structures Technologies for Civil, Mechanical, and Aerospace Systems SPIE.

[6] Kaur N., Li L., Bhalla S., Xia Y., Ni P., Adhikari S. (2017). Integration and Evaluation of Multiple Piezo Configurations for Optimal Health Monitoring of RC Structures. J. Intell. Mater. Syst. Struct..

[7] Baral S., Adhikari S., Negi N., Bhalla S. (2022). Development and Evaluation of Reusable Piezo Sensors for Health Monitoring of Thin-Walled Steel Structures. J. Civ. Struct. Health Monit..

[8] Negi P., Chhabra R., Kaur N., Bhalla S. (2019). Health Monitoring of Reinforced Concrete Structures Under Impact Loading Using Different Piezo-Based Configurations. Constr. Build. Mater..

[9] Negi P., Chakraborty T., Kaur N., Bhalla S. (2018). EMI Effectiveness Investigation on Embedded PZT Patches at Varying Orientations for Monitoring Concrete Hydration. Constr. Build. Mater..

[10] Bhalla S., Moharana S., Talakokula V., Kaur N. (2017). Piezoelectric Materials: Applications in SHM, Energy Harvesting & Biomechanics.

[11] Joshi B., Adhikari S., Bhalla S. (2015). Review on the Use of Piezo Sensors for Structural Health Monitoring, Energy Harvesting and Bio Mechanical Applications. Int. J. Appl. Eng. Res..

[12] Krishnamurthy K., Lalande F., Rogers C.A. Effect of Temperature on the Electrical Impedance of Piezoelectric Sensors. Proceedings of the SPIE North American Conference on Smart Structures and Materials.

[13] Bhalla S. (2001). Smart System Based Automated Health Monitoring of Structures. Master’s Thesis.

[14] Yang Y., Lim Y.Y., Soh C.K. (2008). Practical Issues Related to The Application of Electro-mechanical Impedance Technique in the Structural Health Monitoring of Civil Structures: I. Experiment. Smart Mater. Struct..

[15] Baptista F.G., Budoya D.E., Dealmeida V.A., Ulson J.A.C. (2014). An Experimental Study on the Effect of Temperature on Piezoelectric Sensors for Impedance-Based Structural Health Monitoring. Sensors.

[16] Na W.S., Lee H. (2016). Experimental Investigation for an Isolation Technique on Conducting the Electro-mechanical Impedance Method in High-temperature Pipeline Facilities. J. Sound Vib..

[17] Wandowski T., Malinowski P.H., Ostachowicz W.M. (2016). Temperature and Damage Influence on Electro-mechanical Impedance Method used for Carbon Fibre–reinforced Polymer Panels. J. Intell. Mater. Syst. Struct..

[18] Gianesini B.M., Cortez N.E., Antunes R.A., Filho J.V. (2020). Method for removing temperature effect in impedance-based structural health monitoring systems using polynomial regression. Struct. Health Monit..

[19] Dongyu X., Sourav B., Yanbing W., Shifeng H., Xin C. (2015). Temperature and loading effects of embedded smart piezoelectric sensor for health monitoring of concrete structures. Constr. Build. Mater..

[20] Huynh T.C., Kim J.T. (2017). Quantification of temperature effect on impedance monitoring via PZT interface for prestressed tendon anchorage. Smart Mater. Struct..

[21] Han G., Su Y.-F., Nantung T., Lu N. (2021). Mechanism for using piezoelectric sensor to monitor strength gain process of cementitious materials with the temperature effect. J. Intell. Mater. Syst. Struct..

[22] Antunes R.A., Cortez N.E., Gianesini B.M., Filho J.V. (2019). Modeling, Simulation, Experimentation, and Compensation of Temperature Effect in Impedance-Based SHM Systems Applied to Steel Pipes. Sensors.

[23] Hoshyarmanesh H., Ghodsi M., Kim M., Cho H.H., Park H.-H. (2019). Temperature Effects on Electro-mechanical Response of Deposited Piezoelectric Sensors Used in Structural Health Monitoring of Aerospace Structures. Sensors.

[24] Park G., Kabeya K., Cudney H.H., Inman D.J. (1999). Impedance-based Structural Health Monitoring for Temperature Varying Applications. JSME Int. J..

[25] Giurgiutiu V., Reynolds A., Rogers C.A. (1999). Experimental Investigation of E/M Impedance Health Monitoring for Spot-Welded Structural Joints. J. Intell. Mater. Syst. Struct..

[26] Adhikari S., Bhalla S. (2019). Modified Dual Piezo Configuration for Improved Structural Health Monitoring Using Electro Mechanical Impedance (EMI) Technique. Exp. Tech..

[27] Adhikari S. (2015). Dual Piezo System for Structural Health Monitoring Using EMI Technique. Master’s Thesis.

[28] (2019). PI Ceramic, “Product Catalogue”, Lindenstrabe, Germany. http://www.piceramic.de.

[29] Lipski A., Mrozinski S. (2012). The Effects of Temperature on the Strength Properties of Aluminum Alloy 2024-T3. Acta Mech. Autom..

[30] Narender K., Rao AS M., Rao KG K., Krishna N.G. (2013). Temperature Dependence of Density and Thermal Expansion of Wrought Aluminum Alloys 7041, 7075 and 7095 by Gamma Ray Attenuation Method. J. Mod. Phys..

[31] (2019). Agilent Technologies “Agilent VEE pro Quick Start Guide”. http://www.agilent.com.

